# The khmer software package: enabling efficient nucleotide sequence analysis

**DOI:** 10.12688/f1000research.6924.1

**Published:** 2015-09-25

**Authors:** Michael R. Crusoe, Hussien F. Alameldin, Sherine Awad, Elmar Boucher, Adam Caldwell, Reed Cartwright, Amanda Charbonneau, Bede Constantinides, Greg Edvenson, Scott Fay, Jacob Fenton, Thomas Fenzl, Jordan Fish, Leonor Garcia-Gutierrez, Phillip Garland, Jonathan Gluck, Iván González, Sarah Guermond, Jiarong Guo, Aditi Gupta, Joshua R. Herr, Adina Howe, Alex Hyer, Andreas Härpfer, Luiz Irber, Rhys Kidd, David Lin, Justin Lippi, Tamer Mansour, Pamela McA'Nulty, Eric McDonald, Jessica Mizzi, Kevin D. Murray, Joshua R. Nahum, Kaben Nanlohy, Alexander Johan Nederbragt, Humberto Ortiz-Zuazaga, Jeramia Ory, Jason Pell, Charles Pepe-Ranney, Zachary N. Russ, Erich Schwarz, Camille Scott, Josiah Seaman, Scott Sievert, Jared Simpson, Connor T. Skennerton, James Spencer, Ramakrishnan Srinivasan, Daniel Standage, James A. Stapleton, Susan R. Steinman, Joe Stein, Benjamin Taylor, Will Trimble, Heather L. Wiencko, Michael Wright, Brian Wyss, Qingpeng Zhang, en zyme, C. Titus Brown

**Affiliations:** 1 Microbiology and Molecular Genetics, Michigan State University, East Lansing, MI, USA; 2 Department of Plant, Soil and Microbial Sciences, Michigan State University, East Lansing, MI, USA; 3 Population Health and Reproduction, University of California, Davis, Davis, CA, USA; 4 Department of Biomedical Engineering, Oregon Health and Science University, Portland, OR, USA; 5 Biology Department, San Jose State University, San Jose, CA, USA; 6 School of Life Sciences and The Biodesign Institute, Arizona State University, Tempe, AZ, USA; 7 Genetics, Michigan State University, East Lansing, MI, USA; 8 Computational and Evolutionary Biology, Faculty of Life Sciences, University of Manchester, Manchester, UK; 9 Micron Technology, Seattle, WA, USA; 10 Invitae, San Francisco, CA, USA; 11 Computer Science and Engineering, Michigan State University, East Lansing, MI, USA; 12 Independent Researcher, Munich, Germany; 13 Mathematics Institute, University of Warwick, Warwick, UK; 14 Eastlake Data, Seattle, WA, USA; 15 Graduate Program, University of Maryland, College Park, MD, USA; 16 Athinoula A. Martinos Center for Biomedical Imaging, Department of Radiology, Massachusetts General Hospital, Charlestown, MA, USA; 17 Independent Researcher, Seattle, WA, USA; 18 Center for Microbial Ecology, Michigan State University, East Lansing, MI, USA; 19 Department of Agricultural and Biosystems Engineering, Iowa State University, Ames, IA, USA; 20 Department of Biology, University of Utah, Salt Lake City, UT, USA; 21 ConSol* Software GmbH, Munchen, Germany; 22 Independent Researcher, Sydney, Australia; 23 Verdematics, Fremont, CA, USA; 24 Independent Researcher, San Francisco, CA, USA; 25 Clinical Pathology, Mansoura University, Mansoura, Egypt; 26 Addgene, Cambridge, MA, USA; 27 Biochemistry and Molecular Biology, Michigan State University, East Lansing, MI, USA; 28 ARC Centre of Excellence in Plant Energy Biology, The Australian National University, Canberra, ACT, Australia; 29 BEACON Center, Michigan State University, East Lansing, MI, USA; 30 Independent Researcher, New Orleans, LA, USA; 31 Centre for Ecological and Evolutionary Synthesis, Dept. of Biosciences, University of Oslo, Oslo, Norway; 32 Department of Computer Science, Rio Piedras Campus, University of Puerto Rico, San Juan, Puerto Rico; 33 Biochemistry, St. Louis College of Pharmacy, St. Louis, MO, USA; 34 Crop and Soil Sciences, Cornell University, Ithaca, NY, USA; 35 Department of Bioengineering, UC Berkeley, Berkeley, CA, USA; 36 Department of Molecular Biology and Genetics, Cornell University, Ithaca, NY, USA; 37 Data Visualization, Newline Technical Innovations, Windsor, CO, USA; 38 Electrical and Computer Engineering, University of Minnesota, Minneapolis, MN, USA; 39 Ontario Institute for Cancer Research, Toronto, ON, Canada; 40 Computer Science, University of Toronto, Toronto, ON, Canada; 41 Division of Geological and Planetary Sciences, California Institute of Technology, Pasadena, CA, USA; 42 Dept of Physics and Dept of Materials, Imperial College London, London, UK; 43 Genetics and Genomic Sciences, Icahn School of Medicine at Mount Sinai, New York, NY, USA; 44 Department of Biology, Indiana University, Bloomington, IN, USA; 45 Bioinformatics and Computational Biology Graduate Program, Iowa State University, Ames, IA, USA; 46 Chemical Engineering & Materials Science, Michigan State University, East Lansing, MIS, USA; 47 The New York Eye and Ear Infirmary of Mount Sinai, New York, NY, USA; 48 Independent Researcher, Providence, RI, USA; 49 Mathematics and Computer Science Division, Argonne National Laboratory, Lemont, IL, USA; 50 Department of Genetics, Smurfit Institute, Trinity College Dublin, Dublin, Ireland; 51 Independent Researcher, Boston, MA, USA

**Keywords:** bioinformatics, dna sequencing analysis, k-mer, kmer, khmer, online, low-memory, streaming

## Abstract

The khmer package is a freely available software library for working efficiently with fixed length DNA words, or k-mers. khmer provides implementations of a probabilistic k-mer counting data structure, a compressible De Bruijn graph representation, De Bruijn graph partitioning, and digital normalization. khmer is implemented in C++ and Python, and is freely available under the BSD license at https://github.com/dib-lab/khmer/.

## Introduction

DNA words of a fixed-length k, or “k-mers”, are a common abstraction in DNA sequence analysis that enable alignment-free sequence analysis and comparison. With the advent of second-generation sequencing and the widespread adoption of De Bruijn graph-based assemblers, k-mers have become even more widely used in recent years. However, the dramatically increased rate of sequence data generation from Illumina sequencers continues to challenge the basic data structures and algorithms for k-mer storage and manipulation. This has led to the development of a wide range of data structures and algorithms that explore possible improvements to k-mer-based approaches.

Here we present version 2.0 of the khmer software package, a high-performance library implementing memory- and time-efficient algorithms for the manipulation and analysis of short-read data sets. khmer contains reference implementations of several approaches, including a probabilistic k-mer counter based on the CountMin Sketch ^1^, a compressible De Bruijn graph representation built on top of Bloom filters ^2^, a streaming lossy compression approach for short-read data sets termed “digital normalization” ^3^, and a generalized semi-streaming approach for k-mer spectral analysis of variable-coverage shotgun sequencing data sets ^4^.

khmer is both research software and a software product for users: it has been used in the development of novel data structures and algorithms, and it is also immediately useful for certain kinds of data analysis (discussed below). We continue to develop research extensions while maintaining existing functionality.

The khmer software consists of a core library implemented in C++, a CPython library wrapper implemented in C, and a set of Python “driver” scripts that make use of the library to perform various sequence analysis tasks. The software is currently developed on GitHub under https://github.com/dib-lab/khmer, and it is released under the BSD License. There is greater than 87% statement coverage under automated tests, measured on both C++ and Python code but primarily executed at the Python level.

## Methods

### Implementation

The core data k-mer counting data structures and graph traversal code are implemented in C++, and then wrapped for Python in hand-written C code, for a total of 10.5k lines of C/C++ code. The command-line API and all of the tests are written in 13.7k lines of Python code. C++ FASTQ and FASTA parsers came from the SeqAn library ^5^.

Documentation is written in reStructuredText, compiled with Sphinx, and hosted on ReadTheDocs.org.

We develop khmer on github.com as a community open source project focused on sustainable software development ^6^, and encourage contributions of any kind. As an outcome of several community events, we have comprehensive documentation on contributing to khmer at https://khmer.readthedocs.org/en/latest/dev/ ^7^. Most development decisions are discussed and documented publicly as they happen.

### Operation

khmer is primarily developed on Linux for Python 2.7 and 64-bit processors, and several core developers use Mac OS X. The project is tested regularly using the Jenkins continuous integration system running on Ubuntu 14.04 LTS and Mac OS X 10.10; the current development branch is also tested under Python 3.3, 3.4, and 3.5. Releases are tested against many Linux distributions, including RedHat Enterprise Linux, Debian, Fedora, and Ubuntu. khmer should work on most UNIX derivatives with little modification. Windows is explicitly not supported.

Memory requirements for using khmer vary with the complexity of data and are user configurable. Several core data structures can trade memory for false positives, and we have explored these details in several papers, most notably Pell *et al.* 2012 ^2^ and Zhang *et al.* 2014 ^1^. For example, most single organism mRNAseq data sets can be processed in under 16 GB of RAM ^3, 8^, while memory requirements for metagenome data sets may vary from dozens of gigabytes to terabytes of RAM.

The user interface for khmer is via the command line. The command line interface consists of approximately 25 Python scripts; they are documented at http://khmer.readthedocs.org/ under User Documentation. Changes to the interface are managed with semantic versioning ^9^ which guarantees command line compatibility between releases with the same major version.

khmer also has an unstable developer interface via its Python and C++ libraries, on which the command line scripts are built.

## Use cases

khmer has several complementary feature sets, all centered on short-read manipulation and filtering. The most common use of khmer is for preprocessing short read Illumina data sets prior to *de novo* sequence assembly, with the goals of decreasing compute requirements for the assembler as well as potentially improving the assembly results.

### Prefiltering sequence data for *de novo* assembly with digital normalization

We provide an implementation of a novel streaming “lossy compression” algorithm in khmer that performs abundance normalization of shotgun sequence data. This “digital normalization” algorithm eliminates redundant short reads while retaining sufficient information to generate a contig assembly ^3^. The algorithm takes advantage of the online k-mer counting functionality in khmer to estimate per-read coverage as reads are examined; reads can then be accepted as novel or rejected as redundant. This is a form of error reduction, because the net effect is to decrease not only the total number of reads considered for assembly, but also the total number of errors considered by the assembler. Digital normalization results in a decrease of the amount of memory needed for *de novo* assembly of high-coverage data sets with little to no change in the assembled contigs.

Digital normalization is implemented in the script normalize-by-median.py. This script takes as input a list of FASTA or FASTQ files, which it then filters by abundance as described above; see 3 for details. The output of the digital normalization script is a downsampled set of reads, with no modifications to the individual reads. The three key parameters for the script are the k-mer size, the desired coverage level, and the amount of memory to be used for k-mer counting. The interaction between these three parameters and the filtering process is complex and depends on the data set being processed, but higher coverage levels and longer k-mer sizes result in less data being removed. Lower memory allocation increases the rate at which reads are removed due to erroneous estimates of their abundance, but this process is very robust in practice ^1^.

The output of normalize-by-median.py can be assembled using a *de novo* assembler such as Velvet ^10^, IDBA ^11^, Trinity ^12^ or SPAdes ^13^.

### K-mer counting and read trimming

Using a memory-efficient CountMin Sketch data structure, khmer provides an interface for online counting of k-mers in streams of reads. The basic functionality includes calculating the k-mer frequency spectrum in sequence data sets and trimming reads at low-abundance k-mers. This functionality is explored and benchmarked in ^1^.

Basic read trimming is performed by the script filter-abund.py, which takes as arguments a k-mer countgraph (created by khmer’s load-into-counting.py script) and one or more sequence data files. The script examines each sequence to find k-mers below the given abundance cutoff, and truncates the sequence at the first such k-mer. This truncates reads at the location of substitution errors produced by the sequencing process. When processing sequences from variable coverage data sets, filter-abund.py can also be configured to ignore reads that have low estimated abundance.

K-mer abundance distributions can be calculated using the script abundance-dist.py, which takes as arguments a k-mer countgraph, a sequence data file, and an output filename. This script determines the abundance of each distinct k-mer in the data file according to the k-mer countgraph, and summarizes the abundances in a histogram output.

We recently extended digital normalization to provide a generalized semi-streaming approach for k-mer spectral analysis ^4^. Here, we examine read coverage on a per-locus basis in the De Bruijn graph and, once a particular locus has sufficient coverage, call errors or trim bases for all following reads belonging to that graph locus. The approach is “semi-streaming” ^4^ because some reads must be examined twice. This semi-streaming approach enables few-pass analysis of high coverage data sets. More, the approach also makes it possible to apply k-mer spectral analysis to data sets with uneven coverage such as metagenomes, transcriptomes, and whole-genome amplified samples.

Because our core data structure sizes are preallocated based on estimates of the unique k-mer content of the data, we also provide fast and low-memory k-mer cardinality estimation via the script unique-kmers.py. This script uses the HyperLogLog algorithm to provide a probabilistic estimate of the number of unique k-mers in a data set with a guaranteed upper bound ^14^. A manuscript on this implementation is in progress (Irber and Brown, unpublished).

### Partitioning reads into disconnected assembly graphs

We have also built a De Bruijn graph representation on top of a Bloom filter, and implemented this in khmer. The primary use for this so far has been to enable memory efficient *graph partitioning*, in which reads contributing to disconnected subgraphs are placed into different files. This can lead to an approximately 20-fold decrease in the amount of memory needed for metagenome assembly ^2^, and may also separate reads into species-specific bins ^15^.

### Reformatting collections of short reads

In support of the streaming nature of this project, our preferred paired-read format is with pairs interleaved in a single file. As an extension of this, we automatically support a “broken-paired” read format where orphaned reads and pairs coexist in a single file. This enables single input/output streaming connections between tools, while leaving our tools compatible with fully paired read files as well as files containing only orphaned reads.

For converting to and from this format, we supply the scripts extract-paired-reads.py, interleave-reads.py, and split-paired-reads.py to respectively extract fully paired reads from sequence files, interleave two files containing read pairs, and split an interleaved file into two files containing read pairs.

In addition, we supply several utility scripts that we use in our own work. These include sample-reads-randomly.py for performing reservoir sampling of reads and readstats.py for summarizing sequence files.

## Summary

The khmer project is an increasingly mature open source scientific software project that provides several efficient data structures and algorithms for analyzing short-read nucleotide sequencing data. khmer emphasizes online analysis, low memory data structures and streaming algorithms. khmer continues to be useful for both advancing bioinformatics research and analyzing biological data.

## Software availability

### Software available from

https://khmer.readthedocs.org/en/v2.0/

### Link to source code

https://github.com/dib-lab/khmer/releases/tag/v2.0

### Link to archived source code as at time of publication

http://dx.doi.org/10.5281/zenodo.31258 ^16^

### Software license

Michael Crusoe: Copyright: 2010–2015, Michigan State University. Copyright: 2015, The Regents of the University of California. Redistribution and use in source and binary forms, with or without modification, are permitted provided that the following conditions are met:

- Redistributions of source code must retain the above copyright notice, this list of conditions and the following disclaimer.
- Redistributions in binary form must reproduce the above copyright notice, this list of conditions and the following disclaimer in the documentation and/or other materials provided with the distribution.
- Neither the name of the Michigan State University nor the names of its contributors may be used to endorse or promote products derived from this software without specific prior written permission.

THIS SOFTWARE IS PROVIDED BY THE COPYRIGHT HOLDERS AND CONTRIBUTORS "AS IS" AND ANY EXPRESS OR IMPLIED WARRANTIES, INCLUDING, BUT NOT LIMITED TO, THE IMPLIED WARRANTIES OF MERCHANTABILITY AND FITNESS FOR A PARTICULAR PURPOSE ARE DISCLAIMED. IN NO EVENT SHALL THE COPYRIGHT HOLDER OR CONTRIBUTORS BE LIABLE FOR ANY DIRECT, INDIRECT, INCIDENTAL, SPECIAL, EXEMPLARY, OR CONSEQUENTIAL DAMAGES (INCLUDING, BUT NOT LIMITED TO, PROCUREMENT OF SUBSTITUTE GOODS OR SERVICES; LOSS OF USE, DATA, OR PROFITS; OR BUSINESS INTERRUPTION) HOWEVER CAUSED AND ON ANY THEORY OF LIABILITY, WHETHER IN CONTRACT, STRICT LIABILITY, OR TORT (INCLUDING NEGLIGENCE OR OTHERWISE) ARISING IN ANY WAY OUT OF THE USE OF THIS SOFTWARE, EVEN IF ADVISED OF THE POSSIBILITY OF SUCH DAMAGE.

## References

[1] Zhang Q Pell J Canino-Koning R: These are not the k-mers you are looking for: Efficient online k-mer counting using a probabilistic data structure. *PLoS One.* 2014;9(7):e101271. 10.1371/journal.pone.0101271 25062443 PMC4111482

[2] Pell J Hintze A Canino-Koning R: Scaling metagenome sequence assembly with probabilistic de Bruijn graphs. *Proc Natl Acad Sci U S A.* 2012;109(33):13272–7. 10.1073/pnas.1121464109 22847406 PMC3421212

[3] Brown CT Howe A Zhang Q: A reference-free algorithm for computational normalization of shotgun sequencing data. arXiv preprint.2012 Reference Source

[4] Zhang Q Awad S Brown CT: Crossing the streams: a framework for streaming analysis of short DNA sequencing reads. *PeerJ PrePrints.* 2015;3:e1100 10.7287/peerj.preprints.890v1

[5] Döring A Weese D Rausch T: SeqAn an efficient, generic C++ library for sequence analysis. *BMC Bioinformatics.* 2008;9(1):11. 10.1186/1471-2105-9-11 18184432 PMC2246154

[6] Crusoe MR Brown CT: Walking the talk: adopting and adapting sustainable scientific software development processes in a small biology lab. *figshare.* 2013 10.6084/m9.figshare.791567 PMC5142744 27942385

[7] Brown CT Crusoe MR: Channeling community contributions to scientific software: a sprint experience. *figshare.* 2014 10.6084/m9.figshare.1112541 PMC5104275 27840675

[8] Lowe EK Swalla BJ Brown CT: Evaluating a lightweight transcriptome assembly pipeline on two closely related ascidian species. *PeerJ Preprints.* 2014;2 10.7287/peerj.preprints.505v1

[9] Preston-Werner T: Semantic versioning 2.0.0.2015 [Online; accessed 3-August-2015]. Reference Source

[10] Zerbino DR Birney E: Velvet: algorithms for *de novo* short read assembly using de Bruijn graphs. *Genome Res.* 2008;18(5):821–9. 10.1101/gr.074492.107 18349386 PMC2336801

[11] Peng Y Leung HCM Yiu SM: IDBA–a practical iterative de Bruijn graph *de novo* assembler. In *Research in Computational Molecular Biology* 2010;426–440. 10.1007/978-3-642-12683-3_28

[12] Haas BJ Papanicolaou A Yassour M: *De novo* transcript sequence reconstruction from RNA-seq using the Trinity platform for reference generation and analysis. *Nat Protoc.* 2013;8(8):1494–512. 10.1038/nprot.2013.084 23845962 PMC3875132

[13] Bankevich A Nurk S Antipov D: SPAdes: a new genome assembly algorithm and its applications to single-cell sequencing. *J Comput Biol.* 2012;19(5):455–477. 10.1089/cmb.2012.0021 22506599 PMC3342519

[14] Flajolet P Fusy E Gandouet O: HyperLogLog: the analysis of a near-optimal cardinality estimation algorithm. *DMTCS Proceedings.* 2008; (1). Reference Source

[15] Howe AC Jansson JK Malfatti SA: Tackling soil diversity with the assembly of large, complex metagenomes. *Proc Natl Acad Sci U S A.* 2014;111(13):4904–9. 10.1073/pnas.1402564111 24632729 PMC3977251

[16] Crusoe MR Alameldin HF Awad S: The khmer project v2.0. *Zenodo.* 2015 Data Source

