# Supplementary material for: The khmer software package: enabling efficient nucleotide sequence analysis
Source: F1000Res. 2015 Sep 25;4:900. [Version 1] doi: 10.12688/f1000research.6924.1 (PMC4608353; doi:10.12688/f1000research.6924.1)
Supplement: Peer review history

# Peer review: Referee response for version 1

**DOI:** 10.5256/f1000research.7456.r10508

**Published:** 2015-10-08

**Authors:** Rob Patro

**Affiliations:** 1 Computer Science Department, Stony Brook University, Stony Brook, NY, USA

This paper describes version 2 of the khmer software suite.  The software is developed to provide both a set of directly usable tools (e.g. normalize-by-median for digital normalization) as well as an experimental framework for developers looking to design new algorithms and methods.  It has proven very useful on both of these fronts.  The repository is highly watched and starred on GitHub, the developers are very responsive (see more below), and both the senior author's group and other researches seem to be leveraging this framework to build new tools and algorithms.

The paper itself does a good job of describing the software at a high level, including the overall design and goals.  I would have appreciated slightly more detail about the motivation behind the design decisions, and the tradeoffs they entail (e.g. Why have a Python front-end? Why use hand-written binding code rather than a binding generator, like SWIG, that would allow interfaces to other languages as well?).  I understand that a comprehensive description is not feasible in a manuscript of this length.  It would be very interesting to know, however, the cost paid for using the high-level interface rather than the C++ library directly.  When the underlying computation is trivial, simply having to iterate over an enormous number of things in Python could add non-trivial overhead.  Despite these desiderata, I find that the paper is generally well written and does a good job of describing what a new user might want to know about khmer, and so I approve of this manuscript.

Like Daniel, I also downloaded and built the software using the instructions provided in the ReadTheDocs documentation.  The process was simple, and worked well, with the exception of a minor glitch running the tests.  After debugging the cause of the problem, I posted an issue to the GitHub repository, and received a response in less than a day.  I bring this up because, while not an aspect of the paper itself, good developer support is crucial to the long-term survival and utility of a software package — khmer seems to have this.

This brings me to my final point, about the (currently) controversial authorship policy on this paper, which is ancillary to the quality of the paper (and software) itself.  At this point, *I must reserve judgement* on whether I think the authorship policy adopted by this paper is "good" or "bad" (for science, the community, etc.).  Incidentally, this is a dichotomy that does not capture the subtlety or importance of this issue well.  In the manuscript, the authors state "We develop khmer on github.com as a community open source project focused on sustainable software development, and encourage contributions of any kind."  Thus, contributions to khmer are of a potentially wide variety in character (and also, I believe, not simply related to improving or maintaining the code).  Those who contribute to the design, improve the usability, work on documentation, support new and existing users, and develop and propagate best practices are all contributing something valuable to the khmer software "ecosystem".  It is unreasonable to expect a piece of software that is ~25k lines of code (and growing) to be actively developed, maintained, and supported by only a small contingent of people, many of whom may be graduate students soon to graduate and move on.  Thus, if we are interested in the long-term viability and quality of such software, we must adopt a system of credit that values and recognizes a variety of different types of contribution.  On the other hand, I do share the concern that, in the midst of the current authorship system, bestowing that recognition in the form of authorship may have the adverse effect of diminishing the public perception of the very credit one is trying to grant.  Perhaps there is a solution *along* the lines adopted by this paper, or perhaps something drastically different needs to be considered.

I have read this submission. I believe that I have an appropriate level of expertise to confirm that it is of an acceptable scientific standard.

# Peer review: Referee response for version 1

**DOI:** 10.5256/f1000research.7456.r10513

**Published:** 2015-10-06

**Authors:** Daniel Katz

**Affiliations:** 1 Computation Institute, University of Chicago, Chicago, IL, USA

Regarding the paper, it is a fairly straightforward description of a software package, containing all the things that such a paper should have - a description of the goals, the implemented methods, the hardware and software dependencies (systems on which the software has been tested), some guidance on usage, pointers to the software and documentation, and references.

Regarding the software, I did download and build the software, which seemed to work, other than a fair number of warnings.  I was not able to successfully test the software, however, due to issues in https://khmer.readthedocs.org/en/v2.0/user/install.html#run-the-tests  Does this mean I should not approve the article?  Or should I ask the authors for help in understanding the error and hold off on submitting this report?

I would have liked to have chosen "Approved with reservations" for the status of this review, but my reservations are with the F1000 system for this type of paper, not with this specific paper, so in fairness to the authors, given the lack of clarity of what I should be doing as a reviewer for a software paper, I approve this paper based on its quality as a good description of the software, and not on the quality of software (and related documentation) itself.

I have read this submission. I believe that I have an appropriate level of expertise to confirm that it is of an acceptable scientific standard.

# Response

**Published:** 2015-10-06

**Authors:** Daniel S. Katz

**Affiliations:** University of Chicago, USA

In addition to my report, regarding software papers in general under F1000, I believe that much more should be required from their reviewers, and what **is required** should be made clear.  Software journals (e.g., Ubiquity Press's Journal of Open Research Software, Elsevier's Software X) have specific statements of what a reviewer should do, which say a lot about the quality of the review.  For JORS, this is defined on a web page ( http://openresearchsoftware.metajnl.com/about/editorialPolicies/).  For Software X, the criteria are not on the web (as far as I know) but are embedded in the review form/process, and are roughly equivalent.

# Peer review: Referee response for version 1

**DOI:** 10.5256/f1000research.7456.r10514

**Published:** 2015-10-05

**Authors:** Ewan Birney

**Affiliations:** 1 European Molecular Biology Laboratory, European Bioinformatics Institute, Hinxton, UK

This is an update of a widely used tool, khmer, which is in broad use in the technical community around de Bruijn graphs and short reads, based on Bloom filters. It is a good update, provides link to the code, and is sensibly written with tests. I have no concern about the scientific aspect of this paper.

I do find the author inclusion list taking a concept and going to the extreme, and I don't think it is sensible to have an anonymised author (en zyme) on the list, with in effect no way to attribute to a person this. Science's openness in publication is also about attribution. Although I understand Titus' consistency of having all git committers as authors, I think it is sensible to make a distinction of substantial/scientific changes, of which the vast majority of the authors are. Acknowledgements are precisely there to handle these other cases. 

I believe it is uncontroversial to appropriately trim the author list, to use the acknowledgements for anonymous improvement (happens regularly in science) and small details (again, a commonplace practice).

I have read this submission. I believe that I have an appropriate level of expertise to confirm that it is of an acceptable scientific standard, however I have significant reservations, as outlined above.
